# The role of notch signaling pathway in cancer: mechanistic insights, therapeutic potential, and clinical progress

**DOI:** 10.3389/fimmu.2025.1567524

**Published:** 2025-06-24

**Authors:** Haokun Zhang, Wang Hang, ZhaoHui Jing, Bingyan Liu, Xiaoxiao Wang, Yilin Li, Huomin Luo, Huilin Lv, Xingyue Tao, Peter Timashev, Yuzhen Li, Peifeng Li

**Affiliations:** ^1^ College of Food and Bioengineering, Zhengzhou University of Light Industry, Zhengzhou, China; ^2^ Henan Key Laboratory of Cold Chain Food Quality and Safety Control, Zhengzhou University of Light Industry, Zhengzhou, China; ^3^ Institute of Life and Health, Zhengzhou University of Light Industry, Zhengzhou, China; ^4^ Institute for Regenerative Medicine Sechenov First Moscow State Medical University (Sechenov University), Moscow, Russia; ^5^ Basic Medical Department, Graduate School, Chinese People’s Liberation Army General Hospital, Beijing, China

**Keywords:** notch signaling pathway, cancer, tumor development, therapeutic potential, clinical progress

## Abstract

This article presents a comprehensive literature review on the role of the Notch signaling pathway in cancer and its molecular mechanisms. The Notch signaling pathway plays an important role in various types of cancer by regulating key biological processes such as epithelial-endothelial transition, angiogenesis, apoptosis and metabolic reprogramming. This article reviews the relevant mechanisms of the Notch signaling pathway in colorectal cancer, ovarian cancer, oral squamous cell carcinoma, gastric cancer and chronic lymphocytic leukemia, highlighting its dual roles in promoting tumor growth, inhibiting tumor progression and potential therapeutic applications in oncology. Meanwhile, the key roles of the Notch signaling pathway in regulating tumor drug resistance and shaping the tumor microenvironment are discussed, highlighting its importance in clinical applications. Through this review, some ideas and hints for future research directions can be provided to the readers of Notch pathway related research.

## 1 Introduction

The Notch gene was first described and named in the 20th century by the American scientist Dexter J. Colman through the study of the Notch wing mutant in Drosophila melanogaster (1). Subsequently, scientists have gradually recognized the key role of Notch genes in regulating complex physiological processes in a wide range of organisms, and their major functions are thought to be involved in the development of organ morphology, the coordination of tissue function and repair after tissue injury (2). In recent years, due to the rapid development of tumor therapeutic technologies, gene editing tools and bioinformatics methods, there are significant differences in the degree of dependence of different tumor types on the Notch signaling pathway, and the treatment of tumors by the Notch signaling pathway is still unable to overcome the problems of drug resistance and the impact on the function of healthy tissues, which call for an in-depth exploration of the specific mechanisms of the Notch signaling pathway in the development and progression of tumors. These issues call for an in-depth exploration of its specific mechanism of action in tumor development and progression. Therefore, the study of how the Notch pathway regulates tumors may provide a deeper understanding of the complexity and potential therapeutic applications of the Notch pathway and provide a new approach for innovative tumor therapeutic strategies.

## 2 Introduction to the notch signaling pathway

The mammalian Notch signaling pathway consists mainly of four Notch receptors (Notch1-4) (3), five Notch ligands (δ-like ligand 1 (DLL1), δ-like ligand 3 (DLL3), δ-like ligand 4 (DLL4), Jagged-1 (JAG1) and Jagged-2 (JAG2)), and a large number of DNA-binding proteins and downstream target genes (4). Each Notch receptor has a number of DNA-binding proteins and downstream target genes (4), of which alterations in any one can affect Notch signaling, including cell proliferation and differentiation (5).Dysregulation of Notch activity can lead to a variety of diseases, including genetic disorders, cardiovascular disease and cancer (6). Among these, each Notch receptor has its own unique function, of which Notch1 is involved in the regulation of processes such as embryonic development, adult tissue maintenance and immune cell development, and plays a critical role in a wide range of cells (7).Notch2 has a similar role in cell signaling to Notch1, but Notch2 may have a specific role in the regulation of angiogenesis (8). Notch3, on the other hand, has a tumor suppressor role (9). Notch4 function is relatively poorly understood and has been implicated in carcinogenesis (10).

## 3 Molecular mechanism of the notch signaling pathway

The Notch signaling pathways include the classical Notch signaling pathway, which is activated by ligand-receptor binding (11), and the non-classical Notch signaling pathway, which interacts with other signaling pathways in the Notch intracellular structural domain (NICD) and in the nucleus and directly intervenes in the regulation of transcription of target genes (12).

**Figure 1:**
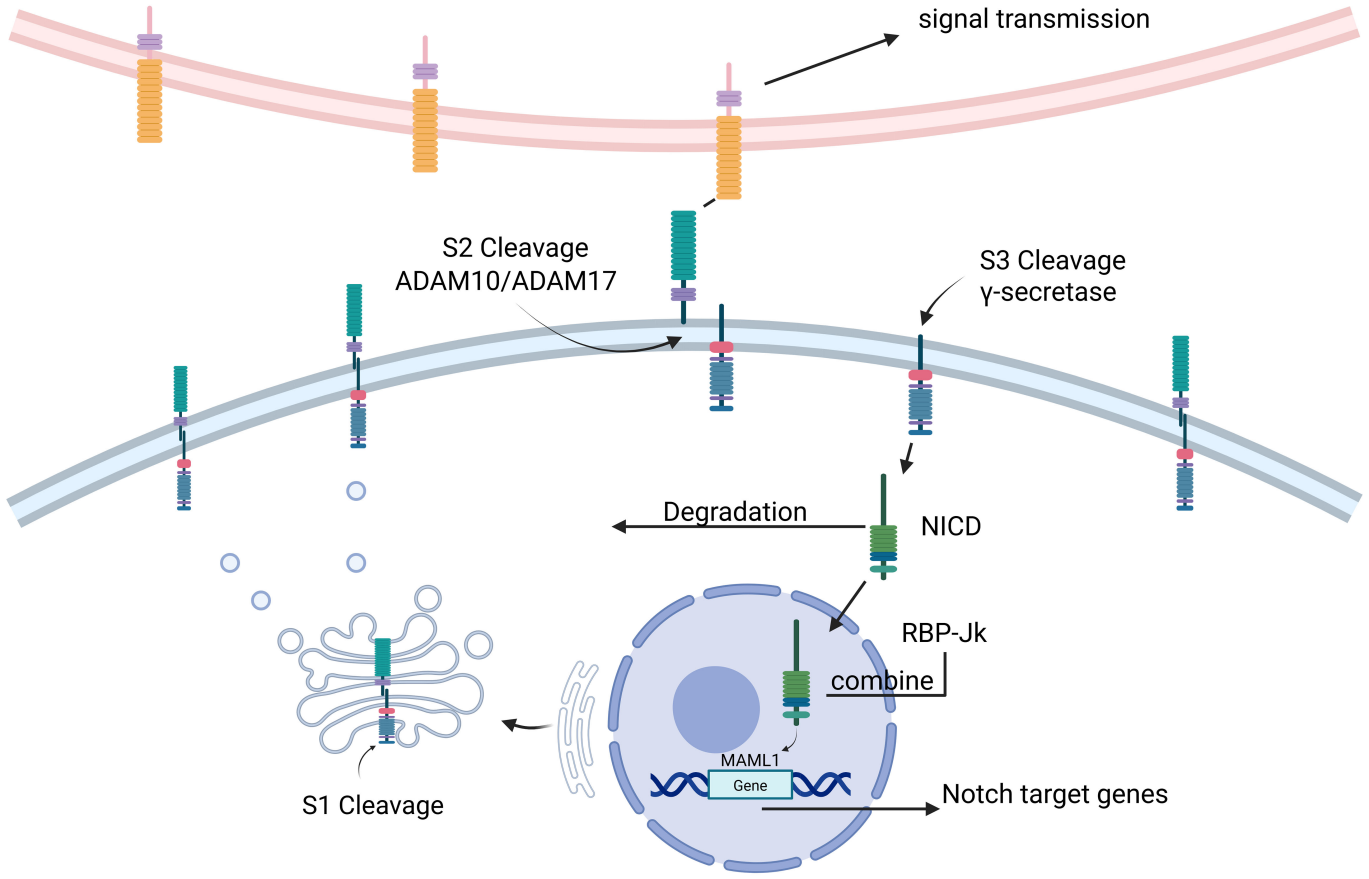
(Figure created using BioRender.com). NICD Notch intracellular domain, ADAM a disintegrin and metalloprotease, MAML mastermind-like. The Notch signaling pathway is where Notch precursors are formed in the endoplasmic reticulum after transcription and translation, translocated to the Golgi for S1 cleavage. The extracellular structural domains of the Notch receptor and ligand are then ubiquitinated by the ligand and initiate endocytosis for S2 cleavage. The Notch receptor then undergoes S3 cleavage by γ-secretase, a process that leads to the release of soluble NICD from Notch. Subsequently, NICD is degraded in the cytoplasm or translocated to the nucleus where it binds to the DNA-binding protein *RBP-Jκ*, which recruits Mastermind-like transcriptional co-activator 1 (MAML1) to form a multiprotein-DNA complex. This complex initiates Notch target genes.

The classical Notch signaling pathway involves the formation of Notch precursors in the endoplasmic reticulum following transcription and translation, leading to the glycosylation of the Notch receptor (13), which is then transported to the Golgi apparatus where it is cleaved into a mature heterodimer in a process also known as S1 cleavage (14). The extracellular structural domain of the Notch receptor and ligand is then ubiquitinated by the ligand, initiating endocytosis and inducing a conformational change in the receptor, exposing the S2 cleavage site of the receptor, which cleaves the extracellular structural domain of Notch and initiates signaling via the metalloproteinases ADAM10 or ADAM17 (15) in a process known as S2 cleavage (16). This is followed by S3 cleavage of the Notch receptor by γ-secretase (17), a process that results in the release of soluble NICD from Notch. NICD is then degraded in the cytoplasm or translocated to the nucleus where it binds to the DNA-binding protein RBP-Jκ, which recruits mastermind-like transcriptional coactivator 1 (MAML1) to form a multiprotein DNA complex. This complex initiates Notch target genes (18) (**Figure 1**).

In addition to the classical Notch pathway, mature Notch receptors on the cell membrane are endocytosed intracellularly under certain circumstances that are not dependent on ligand binding, thereby activating intracellular transduction of Notch signaling, the so-called non-classical Notch pathway (19). Activation of the non-classical pathway has also received increasing attention in cancer research.

## 4 Research on the notch signaling pathway in cancer

**Figure 2:**
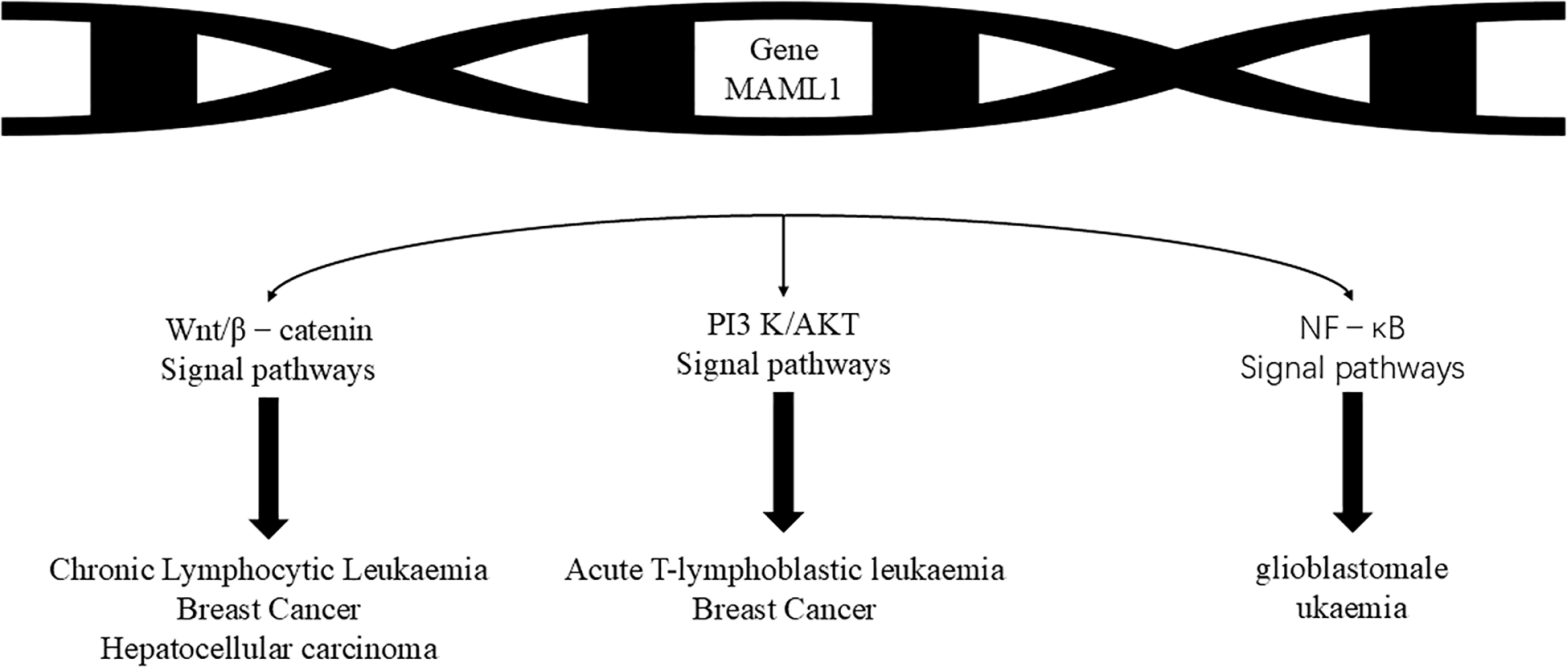
The Notch pathway regulates the following cancer-associated pathways, which then have different functions in different cancers. The MAML1 gene can be used to prevent the development of leukaemia by affecting the Wnt/β-catenin signalling pathway, the PI3 K/AKTS signalling pathway and the NF- kB signaling pathway, leading to chronic lymphocytic leukaemia, breast cancer, hepatocellular carcinoma, acute T-lymphocytic leukaemia, breast cancer and glioblastoma leukaemia.

As mentioned above, the Notch signaling pathway plays a crucial role in regulating the physiological functions of cells and is also closely linked to the development of cancer. It can either promote tumor development or play an oncogenic role in some cancers. In the study of many human cancers, Notch gene mutation or signaling dysregulation can trigger various diseases, such as colorectal cancer (20), prostate cancer (21), glioblastoma and other malignant tumors in which Notch signaling has been found to be downregulated or inactivated. However, Notch plays opposite roles in the same type of cancer. Notch is oncogenic in T-lymphocyte malignancies (e.g. T-ALL) and chronic lymphocytic leukemia, but monostatic in B-cell acute lymphoblastic leukemia, chronic granulomonocytic leukemia and acute myeloid leukemia (22, 23). Notch signaling plays different roles in different cancers and needs to be studied according to the type of cancer(**Figure 2**, **Table 1**).

**Table 1:** Notch signaling in cancer.

Cancer	Notch receptor/ligand	Behave	References
Colorectal cancer	Notch1	Regulating Slug and Snail	(24)
	Jagger-1	Activation of Notch1 signaling	(25)
	NICD1	Increase the number of copies of Notch1	(26)
Ovarian cancer	Notch1	Highly expressed in ovarian cancer	(27)
	Notch 2, Notch 3	Highly expressed in ovarian cancer	(28)
	Jagged2	Highly expressed in ovarian cancer	(29)
	MAML1	Highly expressed in ovarian cancer	(28)
	DLL1	Highly expressed in ovarian cancer	(29)
Oral squamous cell carcinoma	Notch 1, Notch 2	Elevated expression in OSCC	(30)
	Notch 3	Elevated expression in OSCC	(31)
	Notch 4	Elevated expression in OSCC	(31)
	Jagged1	Elevated expression in OSCC	(30)
	NICD	Elevated expression in OSCC	(32)
	DLL4	Elevated expression in OSCC	(33)
Gastric cancer	Notch1	Maintenance of intestinalised epithelial cell proliferation	(34)
	Jagged1	Highly expressed in gastric cancer	(35)
Chronic lymphatic leukaemia	Notch 1	Disrupts the PEST structural domain of the protein	(36)

Notch1 regulation in colorectal cancer Slug and Snail play a direct regulatory role, leading to alterations in E-cadherin, *Jagger-1*-mediated activation of Notch1 signaling, and an increase in Notch1 gene copy number by NICD1. Notch1, Notch 2, Notch 3, *Jagged2*, MAML1, DLL1 were highly expressed in ovarian cancer. Notch 1, Notch 2, Notch 3, Jagged1, NICD, DLL4 in oral squamous cell carcinoma were up-regulated in OSCC cell lines and tissue samples, and Notch 4 expression was elevated in OSCC but low in oral verrucous carcinoma. Notch1 in gastric cancer plays a crucial role in maintaining the continuous proliferation of intestinalised epithelial cells, and the expression of *Jagged1* is proportional to the intensity of invasive aggressiveness. Notch 1 disrupts the PEST structural domain of the protein in chronic lymphatic leukaemia.

### 4.1 The role of notch signaling in colorectal cancer

Colorectal cancer accounts for 1/10 of all cancer cases and deaths and is the third most common cancer in men and the second most common cancer in women, with 1.9 million new colorectal cancer cases and 935,000 deaths in 2020 (37).

In colorectal cancer, the Notch1 signaling pathway plays an important role in the EMT process. In inflammation-induced carcinogenesis, activation of MMP9 can lead to activation of Notch1, which exerts a direct regulatory effect on Slug and Snail, leading to changes in E-cadherin and promoting the EMT process (24). In addition, binding of the Jagged-1 ligand and Notch1 receptor is thought to induce similar mesenchymal transformations, suggesting that Jagged-1-mediated activation of Notch1 signaling plays an important role in inducing the EMT process (25). Colon cancer cells knocked out of Jagged-1 grew much slower than controls, but Jagged-1 deletion is important in promoting colorectal carcinogenesis, a process that induces tumors by inhibiting tumor cell differentiation and promoting angiogenesis (38). Increased Notch1 gene copy number has been found to be significantly associated with poorer survival in patients with advanced colorectal cancer, and tumors with upregulated Notch1 expression in a tumor xenograft model derived from patients with colorectal cancer show significant elevation of Notch1, JAG1 and NICD1, which may be ameliorated by targeting this patient population with Notch1 antibodies (26).

### 4.2 The role of notch signaling in ovarian cancer

The mortality rate for ovarian cancer patients has been 5% in recent years (39). Such a high mortality rate is due to the insidious nature of the disease and the difficulty of early diagnosis, and more than 70% of patients are already at an advanced stage of the disease when they are diagnosed, missing the best time for treatment.

Hopfer et al. (29) published the first literature on the expression of the Notch signaling pathway in ovarian cancer in 2005. The literature found that Jagged2, DLL1, Manic Fringe and TSL were more frequently expressed in ovarian adenocarcinomas, while Deltex and Mastermind were more frequently expressed in adenomas, as detected by RT-PCR.Wang et al. (27) used immunohistochemistry, Western blot and RT-PCR to detect Notch1 expression. It was found that the expression of Notch1 in ovarian cancer tissues was significantly higher than that in matched normal ovarian tissues, while Notch1 was highly expressed in ovarian cancer cell lines A2780 and HO-8910, and the expression of Notch1 gradually increased with the poor differentiation of ovarian cancer tissues and the increase of FIGO stage. It can be seen that for the same factor, the expression level is different in different tumor tissues. High expression of NICD in primary ovarian cancer tumors is an independent poor prognostic factor for overall survival (40). Defreitas et al. (28) used bioinformatics methods to analyses multiple databases from CSIOVDB, PRECOG, GENT and CCLE and systematically showed that Notch2, Notch3 and MAML1 were highly expressed in ovarian cancer and were critical for overall patient survival. In conclusion, Notch1-3, Jagged1 and Hes1 are more highly expressed in highly malignant ovarian cancers, and upregulation of Notch pathway component protein expression in ovarian cancer is associated with shortened overall and disease-free survival, especially in patients with advanced tumors. It is therefore hypothesized that Notch may be used as a marker of poor prognosis.

### 4.3 The role of notch signaling in oral squamous cell carcinoma

Oral squamous cell carcinoma (OSCC) accounts for more than 90% of all oral cancers and is the most common oral and maxillofacial tumor (41). As the ninth most common malignant tumor in the world, OSCC has a high morbidity and mortality rate, and the 5-year survival rate of patients is less than 50% (42). Although large-scale genomic and proteomic analyses in recent years have largely elucidated the molecular biological background of OSCC occurrence and development, few genes or proteins have been identified that can be used for targeted therapy (43).

H et al. (30) found that Notch1, Notch2, Jagged1, HES1 and HEY1 were upregulated in both OSCC cell lines and tissue samples, while NICD accumulated in the nucleus of OSCC cells and Jagged1 was expressed in the cytoplasm. This study also showed that inhibition of the Notch pathway using a γ-secretase inhibitor suppressed OSCC cell growth. Y et al. also found that Notch1 and NICD were specifically upregulated in the invasive front of OSCC, Notch1 expression correlated with the clinical staging of OSCC patients, and TNF-α, an inflammatory cytokine, significantly activated the Notch signaling pathway. The absence of Notch1 expression was associated with cell proliferation in OSCC cell lines and TNF-α-dependent inhibition of invasiveness (32). Whole exome sequencing of OSCC tissue showed that 29.2% of the exome of patients had Notch1 mutations, which were predicted to be inactivating mutations in truncated proteins (44). It has also been shown that mutations in the Abruptex structural domain (C1133Y) of Notch1 promote a cellular oncogenic phenotype by increasing cell proliferation and inducing EMT in OSCC cells (45).The expression of Notch1 was significantly increased in poorly differentiated, stage III, stage IV and lymph node metastasis positive OSCC patients, and overexpression of Notch1, NICD, HES1 and DLL4 predicts poor overall survival in OSCC patients (33). Notch3 and Notch4 have also been found to have increased expression in OSCC, but Notch4 has low expression in oral verrucous carcinoma, so Notch4 has been considered as a suitable prognostic marker to differentiate oral verrucous carcinoma from OSCC (31).

### 4.4 The role of notch signaling in gastric cancer

Gastric cancer is the fourth most common malignant tumour of the digestive tract with global incidence rate, and at the same time, China is one of the high incidence areas of gastric cancer, with 400,000 incidence cases every year, accounting for 42% of the total number of gastric cancer cases in the world. As late stage or postoperative recurrence of gastric cancer cannot be treated by surgery, chemotherapy is the main treatment for patients at present. However, chemotherapy has obvious side effects such as nausea and vomiting, and the effect of chemotherapy is unsatisfactory (46).

Studies have shown that the Notch signalling pathway is involved in the differentiation of normal gastric mucosa and epithelium into small concave glands. However, the Notch pathway and glandular differentiation are also closely related to gastric cancer cells, Notch1, Notch2, Notch3 in Notch receptors and Jagged1 and Jagged2 in Notch ligands have been found in human gastric cancer tissue samples, and each of them is expressed with different significance in physiological activities (47). For example, the expression of Notch1 in precancerous lesions and gastric cancer tissues, such as intestinal epithelial chemotaxis and hyperdifferentiated intestinal-type gastric cancer, was significantly higher than that in normal tissues, suggesting that Notch1 plays a crucial role in promoting the transformation of epithelial chemotaxis in the gastric mucosa and maintaining the continuous proliferation of intestinal chemo epithelial cells (34, 48). In addition, both Notch1 and Jagged1 proteins are considered to be independent prognostic predictors for gastric cancer, and their positive expression suggests an association with poor prognosis (35).Yeh et al. (49) reported that Jagged1 is closely related to the pathogenesis of gastric cancer, and its expression is directly proportional to the strength of invasive aggressiveness, implying that patients with high Jagged1 expression have a lower survival rate.

### 4.5 The role of notch signaling in chronic lymphocytic leukemia

Chronic lymphocytic leukemia (CLL) is a blood disorder characterized by clonal proliferation of mature B lymphocytes in the peripheral blood, bone marrow, spleen and lymph nodes, and is common in middle-aged and older people (50). The incidence of CLL is higher in men than in women, and the clinical course of patients is highly variable (51).

Notch1 is one of the most frequently mutated genes in CLL and is a marker of poor prognosis. Most mutations in Notch1 disrupt the structural domains of the protein’s proline-, glutamate-, serine- and threonine-rich polypeptide sequence (PEST), rendering Notch1 unable to maintain its original ubiquitination and leading to proteasomal degradation of the Notch1 receptor (36). In addition, Notch signaling is enhanced by mutations in the gene encoding the Notch1 ubiquitinate, FBXW7, which affects the ubiquitination of the Notch1 protein (52). Point mutations in the untranslated region at the 3’ end of the Notch1 mRNA (3’UTR) often undergo an aberrant splicing event, which also results in the loss of the Notch1 PEST structural domain (53). Clinically, patients with Notch1 mutations develop a more aggressive disease course and the investigation of drugs targeting the Notch pathway is a major advance in changing the prognosis of patients.

## 5 Investigating the specific mechanisms of notch signaling in cancer

The Notch signaling pathway has a complex role in many aspects of cancer (54). For example, the Notch pathway can play a regulatory role in cancer metabolic reprogramming and the tumor microenvironment (55). In the following sections, we will summaries and highlight the specific mechanisms of Notch signaling mediated tumor initiation and progression in terms of epithelial-mesenchymal transition, endothelial-mesenchymal transition, angiogenesis, apoptosis and metabolic reprogramming (**Table 2**).

**Table 2:** Impact of Notch signaling on specific mechanisms.

Mechanisms	Notch receptor/ligand	Behave	References
Emt	Notch1	positive correlation	(56)
	Jagger-1	positive correlation	(57)
EndMT	Notch 2	negative correlation	(58)
	Notch 3	positive correlation	(59)
	Jagged1	positive correlation	(59)
VEGF	JAG1	positive correlation	(60)
	DLL4	positive correlation	(60)
apoptosis	Notch 1	positive correlation	(61, 62)
	Notch 2	positive correlation	(61, 62)
	Notch 3	negative correlation	(63)
metabolic reprogramming	Notch 1	positive correlation	(64)
	Notch 2	positive correlation	(65)

Notch 1 and Jagger-1 promote emt. Notch 3 and Jagged1 promote endMT, Notch 2 inhibits endMT. jag1, DLL4 positively correlate with VEGF. Notch 1, Notch 2 positively correlate with apoptosis, Notch 3 negatively correlates with apoptosis. Notch 1, Notch 2 was positively correlated with metabolic reprogramming.

### 5.1 The role of notch pathway in EMT

EMT stands for epithelial mesenchymal transition, a biological process originally discovered by Elizabeth Hays in the 1980s (66). In this process, epithelial cells lose their cellular polarity and tight junctions and gain the ability to migrate and invade, transforming into cells with mesenchymal properties (67). EMT plays an important role in embryonic development, tissue repair, organ fibrosis, and tumor invasion and metastasis (68). Regulation of the EMT pathway is a complex molecular event involving a variety of molecular mechanisms, including the Notch signaling pathway.

Studies have shown that Notch1 can regulate the EMT process in tumor cells by cross talking with several EMT-related transcription and growth factors (Snail, Slug, Twist1, and FGF, etc.). Timerman et al. (56) found that: in endothelial cells, Notch1 can promote EMT by upregulating the expression of Snail, resulting in the downregulation of E-calmodulin expression. Chen et al. (57) found that in colorectal cancer, miR-598 inhibits the Notch signaling pathway by suppressing the downstream target gene JAG1, thereby regulating the occurrence of EMT. A detailed study found that the relationship between the Notch pathway and EMT is reflected in its effect on cell adhesion molecules such as E-cadherin (69). Overexpression of Notch ICD increases Snail expression and leads to loss of E-cadherin. Downregulation of intercellular adhesion molecules is one of the hallmark changes during EMT, whereas inhibition of the Notch pathway counteracts the decrease in E-cadherin and Snail expression (70). Overexpression of Notch-1 in prostate cancer promotes prostate cancer cell migration and invasion, whereas downregulation of Notch-1 expression inhibits prostate cancer cell migration and invasion that occur through induction of EMT (71). Thus, the Notch signaling pathway may play a role in regulating the expression of EMT-related genes, which promotes the process of EMT and thus affects the invasive and metastatic behavior of tumors.

### 5.2 The role of notch pathway in endothelial-mesenchymal transition

Endothelial-mesenchymal transition (EndMT), similar to EMT, is a process in which endothelial cells lose their endothelial characteristics and transform into mesenchymal cells when exposed to external stimuli. In recent years, EndMT has been shown to play an important role in disease pathogenesis and has been implicated in the development of cardiovascular disease, organ fibrosis and cancer. While Notch signaling is the main signaling pathway regulating the EndMT process, others include TGF-β/Samd3 signaling pathway, BMP signaling pathway, Wnt signaling pathway, etc., and these signaling pathways cross and influence each other to promote the development of EndMT (72).

RLX inhibited the transformation of cardiac fibroblasts into cardiac myofibroblasts, and the mechanism of action was the Notch-mediated TGF-β signaling pathway (73). In endothelial cells, Sahoo et al. (58) found that Notch2, mRNA and protein expression were downregulated in human pulmonary artery endothelial cells stimulated by hypoxia, and that Notch2 inhibition resulted in an increase in the proliferative and migratory capacity of endothelial cells, and Wang et al. (59) found that hypoxia activated the Notch3 signaling pathway and induced high expression of Notch3, Jagged-1 and HES1, which promoted the transformation of pulmonary artery endothelial cells into mesenchymal cells. Thus, the Notch signaling pathway may play a role in regulating the expression of EndMT-related genes and, by affecting its downstream proteins, may inhibit the mesenchymal transdifferentiating of endothelial cells, leading to a decrease in the expression of epithelial cell markers and an increase in the expression of mesenchymal markers.

### 5.3 The role of notch pathway in angiogenesis

Blood vessels are one of the largest organs in the human body, and vascular endothelial growth factor (VEGF) and its receptor are essential for angiogenesis. Two ligands of the Notch signaling pathway (JAG1 and Dll4) play important roles in angiogenesis. In particular, Dll4, which finely regulates the selection of endothelial cells towards tip versus stem cell differentiation (74), is fundamentally involved in the control of VEGF-induced vascular sprouting. Regulation of the Notch signaling pathway has been shown to promote angiogenesis (75) and this process of Notch signaling is critical for tumor angiogenesis.

Qiu et al. (76) showed that Dll4 was positively correlated with VEGF expression in primary glioblastoma, and Fraser et al. (77) demonstrated that *in vivo* blockade of Dll4 with anti-Dll4 monoclonal antibody in primate ovaries increased luteal vasculature and micro vessel density. Wang et al. (60) found that the expression of Dll4 was positively correlated with VEGFR1; while in ovarian cancer tissues, the expression of Notch1 and VEGFR2 was associated with micro vessel density, with Notch1 expression increased in ovarian tumor tissues. correlation; while in ovarian cancer tissues, the expression of Notch1 and VEGFR2 was correlated with micro vessel density, with Notch1 expression increased in ovarian tumor tissues. In malignant tumors such as lung cancer, the relationship between the Notch pathway and vascular endothelial growth factor (VEGF) is particularly close.Jagged1, as a ligand of the Notch pathway, can significantly upregulate the expression of VEGF and promote tumor neovascularization upon binding to Notch1, which in turn promotes tumor proliferation and metastasis (60). In some cases, activation of Notch signaling can also inhibit VEGF expression, thereby reducing angiogenesis (78). The existence of this bidirectional regulatory mechanism complicates the role of the Notch signaling pathway in tumor angiogenesis, and future studies will further explore the molecular mechanisms behind this balance.

### 5.4 The role of notch pathway in apoptosis

Apoptosis consists mainly of extrinsic and intrinsic pathways. The exogenous pathway, also known as the death receptor pathway, induces apoptosis through transmembrane receptor-mediated activation of the caspase family of aspartate protein hydrolases. The endogenous pathway, also known as the mitochondrial pathway, stimulates the direct generation of intracellular signals that cause structural changes in the mitochondrial membrane, releasing pro-apoptotic substances that induce apoptosis (79). The Notch signaling pathway plays an important role in the regulation of apoptosis in tumor cells. Studies have shown that the role of Notch signaling in apoptosis is different for different types of tumor cells.

In hepatocellular carcinoma, QI et al. (61) found that activation of Notch signaling promoted apoptosis in SMMC7721 hepatocellular carcinoma cells, and WANG et al. (62) found that overexpression of Notch signaling enhanced TEAIL-induced apoptosis in hepatocytes. In lung cancer, KONISHI et al. (63) also reported that inhibition of Notch3 expression promoted apoptosis in NSCLC, and LIN et al. (80) further demonstrated the critical role of the Notch3-Jagged1 axis in lung cancer cell apoptosis. In breast cancer, ZHU et al. (81) reported that Linc-OIP5 affects apoptosis in breast cancer MDA-MB-231 cells. The mechanism of apoptosis is complex, and although more and more studies have elucidated the mechanism of the Notch pathway in regulating apoptosis, the diversity and variability of tumor cells still require further exploration of the key nodes of the Notch pathway affecting apoptosis in tumor cells, so as to effectively utilize the Notch pathway for the treatment of tumors.

### 5.5 The role of notch pathway in metabolic reprogramming

The phenomenon of metabolic reprogramming was first discovered in cancer cells in 1927, referring to the phenomenon that tumor cells abandon oxidative phosphorylation in favor of glycolysis for energy supply when using glucose in an aerobic environment. Today, some scientists refer to the change in cellular energy supply as “metabolic reprogramming” (82).

Notch signaling plays a key role in the metabolic reprogramming of cancer cells. Notch signaling has been reported to regulate reverse electron transfer (RET) through interaction with specific respiratory chain complex I (RC-I) proteins containing electron transport Fe-S clusters and NAD(H)-binding sites, which in turn affects energy metabolism in brain cancer stem cells (64). In a mouse model of renal fibrosis, the Notch2 signaling pathway was found to affect the metabolism of mouse kidney cells by regulating the expression of the mitochondrial transcription factor (ATfam), which in turn affects the development of renal fibrosis in mice (65). Particularly during the glycolytic transition. During this transition in cancer cells, the Notch pathway is active and several genes are directly regulated as transcriptional targets. This regulation mediates the shift in cellular metabolism towards the Warburg effect (83, 84). All of the above studies have demonstrated that the Notch signaling pathway can be involved in the metabolic reprogramming of cells in various models.

## 6 Therapeutic potential of the notch signaling pathway

The Notch signaling pathway is a conserved signaling pathway that plays an important role in the regulation of cell fate, tissue development and tissue homeostasis in multicellular organisms (85). Accumulating evidence suggests that the Notch signaling pathway acts as both an oncogenic factor (86) and a tumor suppressor in a variety of cancers (87). Dysregulation of this pathway promotes epithelial-mesenchymal transition and angiogenesis in malignant tumors, which are closely associated with cancer proliferation, invasion and metastasis. In addition, the Notch signaling pathway is involved in tumor drug resistance and tumor suppression. Therefore, a comprehensive understanding of these biological processes is essential for the development of innovative therapeutic strategies targeting the Notch signaling pathway.

### 6.1 The role of notch pathway in tumor drug resistance

Chemotherapy is one of the commonly used treatments for malignant tumors, but acquired resistance is the main reason that hinders the clinical efficacy of patients and leads to treatment failure. Abnormal expression of the Notch signaling pathway is closely related to the formation of tumor chemoresistance (88). It is related to the inherent heterogeneity of tumor cells and genetic background or genetic changes after chemotherapy, while the specific mechanism of Notch signaling pathway involved in the formation of tumor drug resistance is not very clear, Notch signaling pathway may play a role in tumor drug resistance by participating in the process of CSCs phenotyping, EMT and crosstalk with other signaling pathways.

Regarding the involvement of the Notch signaling pathway in the transformation of drug-resistant EMT in tumors, Wang et al. (89) found that the drug-resistant cell line GR had lower expression of E-cadherin, an epithelial adhesion molecule, and higher expression of mesenchymal markers such as vimentin compared to gemcitabine-sensitive cells of GS from pancreatic cancer. Downregulation of Notch-2 and Jagged-1 by siRNA resulted in increased expression of E-cadherin and downregulation of vimentin and ZEB1 expression, reversing EMT. In a study on the involvement of the Notch signaling pathway in regulating CSC formation in tumor resistance, Capodanno et al. (90) found that the expression of inactive and active forms of the Notch2 receptor and its downstream target gene Hes1 was increased in a 5-FU-resistant human insulinoma cell line CM tumor stem cell microspheres. *In vitro* treatment with DAPT alone did not inhibit the clonogenic ability of insulinoma tumor stem-like cells, whereas the combination of DAPT and 5-FU significantly inhibited their clonogenicity. McAuliffe et al. (91) found that the Notch signaling pathway, in particular the Notch-3 signaling pathway, is particularly important for the maintenance of ovarian cancer CSCs and platinum drug resistance. Overexpression of NICD3 significantly increased the expression of CD44, a key stem cell marker, suggesting that there may be cross-talk between Notch-3 and CD44 to maintain the ovarian cancer stem cell phenotype.

### 6.2 The role of notch pathway in tumor suppression

The theoretical basis for the Notch signaling pathway as a target for cancer therapy stems from the fact that Notch can interact with a variety of signaling pathways to achieve regulation of tumor cell proliferation, apoptosis and differentiation. The Notch signaling pathway not only regulates the differentiation, proliferation and apoptosis of normal cells, but its aberrant activation is also closely associated with the development of a variety of cancers (92). Therefore, it is important to investigate the role of the Notch signaling pathway in tumor suppression.

Lewis et al. (93) found that Notch signaling is activated in T-ALL, promotes the expression of the target gene HES, which inhibits the expression of PTEN, which is downregulated and promotes the activation of PI3K/Akt, thus inhibiting the apoptosis of tumor cells, and when a Notch signaling inhibitor is applied, it promotes the expression of PTEN and reverses the anti-apoptotic effect of PI3K/Akt and induces apoptosis. Studies have shown (94) that activation of the Notch signaling pathway can inhibit the expression of INK4a/ARF gene, promote the degradation of p53 by MDM2, down-regulate the oncogenic effect of p53 and inhibit the apoptosis of tumor cells. Khan et al (95) reported that downregulation of Notch signaling promotes the expression of pro-apoptotic protein Bax, reduces the expression of anti-apoptotic protein Bcl-2, and induces apoptosis in prostate cancer cells. Sustained inhibition of Notch1 activity and activation of Bcl-2, Bcl-XL and caspase-3 in breast cancer MCF-7 and MDA-MB-231 cells reduced and promoted tumor cell apoptosis (96).

## 7 Clinical progress related to the notch signaling pathway

As a highly conserved signaling pathway, Notch signaling plays an important role in various biological processes such as growth, development and tissue repair. Notch signaling can produce different or even opposite biological effects with changes in time and cell type. Therefore, researchers have developed a variety of Notch-targeted therapeutics to target the different stages of the Notch pathway (**Table 3**).

**Table 3:** Notch-targeted therapeutics.

Treatment method	Medicines	Machine	References
S1-cleavage inhibitor	CPA	Inhibition of S1 cleavage	(97)
	Thapsigargin	Inhibition of S1 cleavage	(97)
S2 cleavage inhibitor	ZLDI-8	Inhibition of ADAM17	(98)
	GI254023X	Inhibition of ADAM10	(99)
S3 cleavage inhibitor	GSIs	Inhibition of gamma-secretase	(100, 101)

Cyclopiperazonic acid (CPA) with thapsigargin inhibits S1 cleavage ZLDI-8 inhibits S2 cleavage by inhibiting ADAM17, *GI254023X* inhibits S2 cleavage by inhibiting ADAM-10. Gamma-secretase inhibitors (GSIs) inhibit S3 cleavage by inhibiting gamma-secretase.

Currently, therapies targeting the Notch pathway are being evaluated in clinical trials, with the main drugs targeting S1 cleavage being cycloheximide (CPA) and thapsigargin, which block Notch signaling by inhibiting the activity of myoendoplasmic reticulum Ca2+-ATPases (SERCAs) to prevent translocation of mutant Notch1 to the cell surface (97). Since Notch precursors generated in the ER are glycosylated and translocated to the Golgi for S1 cleavage, a process that requires the involvement of Ca2+, SERCAs are critical for Notch signaling as key cofactors that regulate ATP-dependent calcium pumping (102). Notch ligand-receptor binding in S2 cleavage can be blocked by the metalloproteinase ADAM10/TACE (also known as ADAM17) through the known proteolytic cleavage of Notch extracellular structural domains regulates the rate of Notch1 signaling (S2 cleavage). As a key enzyme in S2 cleavage, inhibition of ADAM targets the entire Notch pathway (103). For example, the Notch/ADAM17 inhibitor ZLDI-8 induces apoptosis in chemotherapy-resistant NSCLC and inhibits migration, invasion and EMT of drug-resistant lung cancer cells (98). The ADAM-10 inhibitor GI254023X may be used as a potential treatment for glioblastoma to inhibit cancer cell growth (99). The extracellular-to-intracellular transduction of Notch signaling in S3 cleavage is dependent on γ-secretase complex-mediated S3 cleavage, suggesting that γ-secretase function is closely related to Notch signaling (100). γ-Secretase inhibitors (GSIs) were first used in clinical trials for the treatment of Alzheimer’s disease (AD), but the trial was discontinued before completion of the phase III trial due to severe Notch-related adverse events (101). However, GSIs are still being widely investigated as cancer therapies in preclinical studies and have shown antitumor activity in a variety of cancers, such as hepatocellular carcinoma, where the use of the γ-secretase inhibitor DAPT or the combination of DAPT with the CD147-directed antibody HAb18 has been shown to be effective in the treatment of human HCC tumors transplanted *in situ* (104).

### 7.1 Therapeutic potential of notch signaling pathway-targeted inhibitors

The use of Notch signaling pathway inhibitors in clinical trials has shown great therapeutic potential, such as inhibition of S1, which reduces Ca2+ in the cellular endoplasmic reticulum, thereby inhibiting protein trafficking and altering translation, translocation and maturation of Notch1 and thus bepridil (105). Inhibition of S2 in HCC reduces Notch NICD release and improves the efficacy of anticancer drugs (106). Inhibition of the Notch pathway by miR-3163 targeting ADAM-17 increases the sensitivity of HCC cells to molecularly targeted drugs (107). The γ-secretase inhibitor DAPT in S3 can effectively treat human HCC tumors transplanted *in situ* (104). Researchers can design a series of Notch-targeted therapeutics for each stage of the Notch signaling pathway, and inhibition of the Notch signaling pathway can effectively control the progression of many cancers. However, because the Notch pathway also plays an important role in normal cells (108), the search for drugs that can effectively inhibit tumors without causing health effects has become a focus of future research. Scientists are currently working to develop more tumor-selective Notch inhibitors to improve therapeutic efficacy and reduce side effects. This will also be a future goal of Notch pathway inhibitors for clinical treatment.

## 8 The application prospect of notch signaling pathway in clinical progress

In summary, this review systematically elucidates the molecular mechanism of the Notch signaling pathway and its characterization in cancer. By integrating and analyzing the existing research, we demonstrate the diversity of the pathway and the complexity of its regulatory mechanisms, which can be exerted in different tissues or in the same tissue at different times. These features highlight the need to focus on key factors such as tissue specificity, prophase changes, downstream molecular effects and impact on normal tissues when studying the Notch pathway in cancer.

With the rapid development of genomics and bioinformatics technologies, scientists are not only able to more accurately identify aberrant expression patterns in the Notch pathway, but also to combine multi-omics data for quantitative analysis, which provides the theoretical basis for personalized clinical treatment strategies. However, overactivation of the Notch pathway is not a single hazard, and its intervention can lead to complex pathological responses, so efficacy must be carefully balanced against toxicity and side effects in practical application.

In addition, current research into the application of the Notch signaling pathway in cancer still faces many challenges, the most prominent of which are how to develop targeted drugs with high specificity and low toxicity, and how to effectively respond to the complex regulatory needs of different cancer types and pathological stages. In the future, we will combine gene editing technology, artificial intelligence algorithms and clinical data to further explore and analyses the potential of the Notch pathway in cancer therapy and provide new ideas for the development of targeted drugs and personalized therapeutic strategies.
